# Development and Validation of Breadcrumbing in Affective-Sexual Relationships (BREAD-ASR) Questionnaire: Introducing a New Online Dating Perpetration

**DOI:** 10.3390/ijerph17249548

**Published:** 2020-12-20

**Authors:** Mª Carmen Rodríguez-García, Verónica V. Márquez-Hernández, Genoveva Granados-Gámez, Gabriel Aguilera-Manrique, Helena Martínez-Puertas, Lorena Gutiérrez-Puertas

**Affiliations:** 1 Department of Nursing, Physiotherapy and Medicine, Faculty of Health Sciences, University of Almería, 04120 Almería, Spain; mrg451@ual.es (M.C.R.-G.); genoveva@ual.es (G.G.-G.); gaguiler@ual.es (G.A.-M.); lgp524@ual.es (L.G.-P.); 2 Research Group of Health Sciences CTS-451, University of Almería, 04120 Almería, Spain; 3 Department of Mathematics, University of Almería, 04120 Almería, Spain; hmp603@ual.es

**Keywords:** breadcrumbing, instrument development, psychometrics, social media use, social networking, affective-sexual relationships

## Abstract

New technologies are changing people’s lifestyles and in turn, their way of relating to and interacting with others. Breadcrumbing is one of the new 2.0 concepts linked to the virtual relationship paradigm. This study aimed to design and psychometrically test the Breadcrumbing in Affective-Sexual Relationships (BREAD-ASR) Questionnaire to explore breadcrumbing perpetration in adolescent relationships online. A total of 247 adolescents participated in a paper-and-pencil survey carried out from March to June 2019 in a high school in southeastern Spain. Psychometric analysis showed a satisfactory content and construct validity for the instrument. The ordinal alpha coefficient was 0.83, indicating the BREAD-ASR questionnaire had good internal consistency. The BREAD-ASR questionnaire constitutes a valid and reliable instrument which can be used by health professionals in screenings for breadcrumbing perpetration and to design effective prevention and intervention programs in the community, which may help and support adolescents and families to deal with new forms of online relationships and perpetration successfully.

## 1. Introduction

People’s lifestyles and their way of relating to and interacting with others are changing due to new technologies [1]. The use of information and communication technologies (ICTs) has increased considerably in recent years [2] becoming the primary form of communication between teenagers in intimate relationships [3].

As a result of new technologies, some studies have noted the emergence of new, previously nonexistent ways to initiate, develop and even end relationships [4]. This is due to the fact that advances in communication technology have allowed billions of people to connect with each other using their mobile phones [5], and even start relationships via their phones using mobile applications [6], text messages, instant messaging, voice messages, email, social networks and so on [7].

### Background

Breadcrumbing, also known as “Hansel and Gretelling” [8], is one of the new emerging concepts linked to the virtual relationship paradigm. This term, which comes from the idea of figuratively throwing someone breadcrumbs, is one of the latest trends in dating, and one with which particular precaution must be taken [9]. It is defined as the act of “leading someone on by contacting them intermittently by phone or social media to keep them interested” [10] with no intention of being in a relationship. As far as it has been studied, the perpetrators of breadcrumbing, known as “breadcrumbers”, have been associated with having narcissistic, egocentric personalities, self-esteem issues or emotional problems [11] as well as the need to attract and hold the attention of others to boost their own self-esteem [12], which previously has been observed in other monitoring behaviors [13]. According to Baker and Carreño [14] these types of monitoring behaviors often seem to be related to immaturity and insecurity (e.g., fear of ending the relationship for good or not wanting to hurt the other person), although, it could also be due to a conscious desire to maintain control over the other person and, in turn, feed their own ego [15].

The modus operandi of the breadcrumber is to deceitfully leave a trail of breadcrumbs along the road to a meeting that will never take place [8]. Literature has associated breadcrumbing perpetration to repeated instances of sporadic communication with no commitment in order to keep victims’ hope and interest of a relationship alive [16]. Breadcrumbers do not want to commit to anyone, but they like the attention and keeping their victims interested, generating a dependency on the feeling of being interesting and appealing in the dating world [17]. Therefore, they want their victims to continue thinking about them, but do not necessarily want a committed relationship [18]. In fact, breadcrumbing perpetrators tend to avoid uncomfortable or negative interactions, such as expressing feelings, because of their fear or inability to commit to another person [7]. Though this type of behavior can happen both online and offline, using social networks and applications are the most common ways of expressing these dangerous behaviors [19]. The risks of online dating are very real [8]. Breadcrumbing perpetration can last for weeks or months and it may be a dangerous practice given its association to the feelings of guilt, anxiety and pain reported by victims [11].

Adolescence is a time in which the contact between peers is amplified and marks the beginning of affective-sexual or sexual-affective relationships, affective and sexual experiences in the form of dating, stable relationships, “hooking up,” etc. [20]. It is a phase of experimentation and learning in terms of ones love life, which includes numerous forms of relationships [21]. Adolescents are the main users of the internet and online social networks [22]. They use these new technologies to keep in contact with their partners as well as to establish, maintain and end relationships, and even to reconnect after a breakup [23]. Social networking not only enables their users to flirt and communicate with their romantic partner but it also can play a central role in the process of relational information seeking [13]. On the other hand, technology use can lead to potentially dangerous social interactions [24] and can place adolescents in situations of vulnerability and helplessness in virtual reality and in real life. It can even have a negative impact on their affective-sexual well-being [25]. Nevertheless, the role that social media may play in romantic relationships is an emerging area of research and the majority of studies have been carried out with college-age young adults than with middle adolescents [26]. Furthermore, due to constantly changing online tools for social connection and dating, few studies have recently explored the new ways in which adolescents use and describe their online use for sexual exploration and relationship-building [27].

Health professionals play a key role as health educators to aid the development and maintenance of healthy habits and lifestyles in the community, including adolescents and families. They raise awareness of health-related problems and the dangers associated with certain behaviors [28]. Consequently, they are essential for dealing with new virtual behaviors and providing support [29]. To be able to assess and intervene appropriately, health professionals working with adolescents in the community, in in-patient or out-patient settings need to understand social media and its role in breadcrumbing perpetration. However, like most emerging concepts, the breadcrumbing phenomenon remains unclear.

Currently, breadcrumbing has captured the attention of the press and the knowledge generated surrounding it thus far has been journalistic in nature, similar to other emerging topics [7]. Little scientific research has been carried out as of yet regarding the phenomenon of breadcrumbing, which constitutes a relevant gap in knowledge. The few published research investigations on this phenomenon focus on the prevalence of breadcrumbing victimization [30,31]. Therefore, the scientific knowledge about the profile of individuals who are most likely to become breadcrumbing perpetrators is limited, making it difficult to develop effective prevention and intervention programs. Research has not examined the possible consequences of breadcrumbing perpetration which might be due to the lack of a specific instrument to measure this phenomenon with reliability and validity. It is essential to create a new instrument that leads further research on the profile of breadcrumbing perpetrators as well as on the consequences of breadcrumbing perpetration on adolescents’ health and wellbeing.

The purpose of the study was to develop and test the psychometric properties of the Breadcrumbing Affective-Sexual Relationships (BREAD-ASR) Questionnaire to explore breadcrumbing perpetration in adolescent relationships.

## 2. Materials and Methods

### 2.1. Design

The study was performed in two phases following an instrument development design [32]. Phase 1 included instrument development and the study of content validity, and phase 2 entailed the psychometric evaluation of the instrument.

#### 2.1.1. Phase 1: Instrument Development

First, the Chadha [33] guidelines were followed for the development of the questionnaire. An exhaustive review of the existing literature on the subject was carried out, after which the items were drafted by the research team. After completing this first stage, a total of 22 items were obtained. Following this, a multidisciplinary team of 12 experts in affective-sexual relationships was formed, consisting of psychologists, sexologists and nurses who evaluated the content of the items in terms of relevance. A Likert-type scale from 1 to 4 was used, with 1 signifying not relevant and 4 highly relevant. The content validity index (CVI) for each item was then calculated. In accordance with that established by Lynn [34], a CVI equal to or >0.80 was considered appropriate. Of the 22 initial items, 17 were ultimately considered sufficiently relevant for inclusion in the final instrument, entitled the Breadcrumbing in Affective-Sexual Relationships (BREAD-ASR) Questionnaire.

Once the final questionnaire had been drafted, the 17-item BREAD-ASR was pilot tested in a focus group of high school students to confirm linguistic comprehension. A sample of 30 volunteers ages 12–18 participated in a group session conducted by the research leader. All participants received the BREAD-ASR for self-completion and, after discussion, they confirmed that the questionnaire was comprehensible and clear.

#### 2.1.2. Phase 2: Psychometric evaluation of the BREAD-ASR

A sample of 247 high school students was used to test the BREAD-ASR’ validity and reliability through participation in a paper-and-pencil survey. Items were formulated using a 5-point Likert-type scale (1 = never; 2 = hardly ever; 3 = sometimes; 4 = very often, and 5 = always). A high score therefore indicates a stronger degree of agreement with the corresponding item. Sociodemographic characteristics of the participants were also collected.

Once the corresponding permissions had been obtained, an institute in the province was contacted for data collection. The lead researcher held a session with the participants in order to collect data, following the schedule offered by the center. First, all participants were informed of the objective of the study and the voluntary nature of their participation. All participants then signed an informed consent form. They provided information about their last relationship and the time frame was measured by months. Completion of the questionnaire lasted between 20 and 25 minutes. Finally, the researcher thanked the students for their participation. The study was carried out from March to June 2019 in a public high school in southeastern Spain.

To explore the BREAD-ASR construct validity, an exploratory factor analysis with principal axis factoring and varimax rotation was performed. Literature supports orthogonal methods tend to be easier to interpret and the most recommended rotation for constructing factorial subscales [35,36,37,38]. A polychoric correlation matrix was used in this study as the most appropriate when all variables are Likert scale [39]. A Kaiser–Meyer–Olkin (KMO) >0.7 and a significant Bartlett’s test were considered as indicators of the appropriateness to conduct an exploratory factor analysis. The number of the factors to be extracted was calculated using Scree plot with parallel analysis. The coefficients of the goodness of fit of the proposed models were tested with root mean squared of the residuals (RMSR), root mean square error approximation (RMSEA) and Tucker–Lewis Index (TLI), considering acceptable values closer to 0 for SMSR (recommended value ≤ 0.05); values up to 0.08 for RMSEA (it is desirable not to exceed a 0.06 threshold) and TLI > 0.9 indicating an acceptable fit.

The BREAD-ASR reliability was explored using ordinal alpha (Oα) coefficient, inter-item correlation, item-total correlation coefficients and variations in the ordinal’s alpha coefficients if items were eliminated. Ordinal alpha is considered the most appropriate coefficient for ordinal-type scales [40] and it is conceptually equivalent to Cronbach’s alpha [41], so the internal consistency is acceptable if the ordinal alpha is greater than 0.70, although under more demanding conditions values between 0.80 and 0.90 are preferred.

### 2.2. Participants

The study population was composed of 651 adolescents enrolled in compulsory secondary education at a high school in southeastern Spain. The required sample size was calculated to be 242 with a population proportion of 50%, a confidence level of 95% and a margin of error of 5%. A sample of 247 high school students participated in the paper-and-pencil survey, gathered through convenience sampling. The inclusion criteria included being a student, being 12 to 18 years old, having a mobile phone, having or having had affective-sexual relationships and having consent from the parents or legal guardians to participate in the study. The exclusion criteria included refusing to participate in the investigation. For the sample size, the recommendations of between 5 and 10 subjects for each item of the questionnaire were met [42].

### 2.3. Ethical Considerations

First, permission was sought from the Department of Nursing, Physiotherapy and Medicine Research Committee at the University (EFM18/19). Following this, the educational center where the research would be conducted was contacted in order to request the relevant permissions, which finally were obtained. As those participating in the study were minors, their parents authorized their participation in the study. However, all participants also signed a written informed consent form prior to completing the questionnaire. Information was provided regarding the objective of the study, together with the confidential nature and anonymous processing of the data. Ethical principles were followed in accordance with the Helsinki Guidelines.

### 2.4. Data Analysis

Results were processed using R (The R Foundation for Statistical Computing, Vienna, Austria) Version 3.6.1 and the R Studio interface (RS Team—RStudio, Inc., Boston, Massachusetts, United States) Version 1.1.463. Firstly, a descriptive analysis of the sociodemographic variables (mean, standard deviation, frequencies and percentages) was performed. The CVI was calculated in order to analyse content validity. The Kaiser–Meyer–Olkin (KMO) and Bartlett’s test of sphericity were conducted to evaluate the factorability prior to carrying out the exploratory factor analysis. A polychoric matrix was used for exploratory factorial analysis (EFA) and reliability calculation. The number of the factors to be extracted was calculated using Scree plot with parallel analysis. EFA was computed through a principal axis method using varimax rotation. The goodness of fit indexes used were root means squared of the residuals (RMSR), root mean square error approximation (RMSEA) and Tucker–Lewis Index (TLI) as exploratory estimates. Ordinal alpha value was obtained in order to estimate the internal consistency of the ordinal items’ response scale. A 95% confidence interval was established, considering values of *p* < 0.05 as statistically significant

## 3. Results

### 3.1. Sociodemographic Characteristics of the Participants

Of the total number of participants (N = 247), 44.9% (n = 111) were female and 55.1% (n = 136) were male. The average age was 14.53 (SD = 1.46) with an age range from 12 to 18 years old. All students were in compulsory secondary education at the high school. A summary of the sociodemographic characteristics of the participants can be seen in Table 1.

### 3.2. Generation of Items and Content Validity

As explained in the study design section, 17 items were obtained having previously removed those which did not obtain a CVI greater than or equal to 0.80. The mean and standard deviation (SD) for each item are shown in Table 2.

### 3.3. Construct Validity

#### Exploratory Factor Analysis

EFA with principal axis factoring (PAF) and varimax rotation was used to explore the construct validity of the BREAD-ASR questionnaire. The adequacy of this analysis was verified using the Kaiser–Meyer–Olkin (KMO) test and the Bartlett sphericity test. The KMO test showed 0.79 which indicated a suitable sample adequacy and the Bartlett’s test of sphericity value was significant (χ^2^ = 487.4581; df = 120; *p* ≤ 0.001), evidencing the appropriateness for an EFA to be conducted.

**Figure 1:**
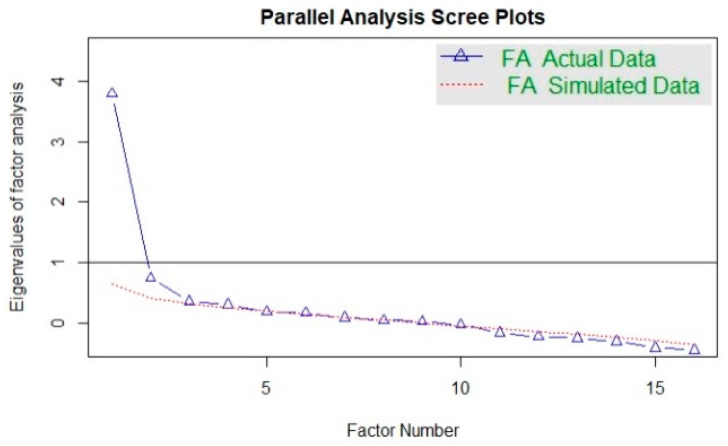
Scree plot.

The number of factors was determined using Scree plot with Parallel Analysis (Figure 1) which suggested that 4 factors would be a good choice.

The four-factor model proposal for the BREAD-ASR questionnaire explained 40% of the variance (see Table 3) and it represented four important dimensions of the measured construct: (1) Communication; (2) Avoidance; (3) Commitment, and (4) Dependence.

Table 4 shows correlations between factors, indicating that the degree of intercorrelation between the variables was very low which confirms the correct selection of the rotation method used.

The goodness-of-fit for the EFA was tested using different fit indices: root mean squared of the residuals (RMSR), root mean squared error of approximation (RMSEA) and Tucker–Lewis Index (TLI), with values indicating adequate fit for the proposed factor model (Table 5).

### 3.4. Reliability

The ordinal alpha coefficient was 0.83, indicating the BREAD-ASR questionnaire had good internal consistency. Furthermore, ordinal alpha was calculated if an item is eliminated to assess the reliability of each item, observing an increased coefficient after removing item 17, therefore, it was eliminated from the instrument. The inter-item correlation coefficients ranged between 0.20 and 0.40, suggesting that the items are reasonably homogenous. The corrected item-total correlation (C-ITC) coefficients were also acceptable in this exploratory study except for item 17, which was finally eliminated (Table 6).

## 4. Discussion

The results of this study contribute to the literature, with the development of the BREAD-ASR questionnaire as a valid and reliable instrument. However, it was not possible to compare the results due to the lack of studies. The complexity of the comparison of the results was also observed in a recent literature review on digital dating violence measures [43]. Therefore, there is an urge to define the construct of new concepts and to agree on instruments to measure the virtual phenomena [22].

In relation to the results of the exploratory factor analysis, four dimensions were extracted: (1) Communication, (2) Avoidance, (3) Commitment and (4) Dependence, which constitute the dimensional structure of the BREAD-ASR questionnaire and help to define the construct of breadcrumbing.

The breadcrumbing phenomenon was associated with repeated instances of sporadic communication without commitment, or breadcrumbs, sufficient to maintain the interest and hope of the other person via social media [16]. This type of communication could slow down the healing process and personal growth of breadcrumbing victims upon deciding the relationship is over, as the perpetrators do not disappear forever. Instead, they are temporarily absent but return after a while, which may worsen the pain of ending the relationship [44]. This contact has also been related to increased feelings of stress about the end of the relationship, more negative feelings, a greater sexual longing for the ex-partner and poorer personal growth after the break up [13].

The lack of commitment along with avoidance seem to be common in other virtual relationships (e.g., ghosting). Perpetrators resort to avoidance when faced with uncomfortable or negative interactions, such as expressing feelings, because of their fear or inability to commit to another person [7]. They are also likely to ignore or delay responding to messages through social networking sites [45]. These variables are measured in the BREAD-ASR questionnaire.

Regarding commitment, numerous forms of relationships have emerged from the online context [21]. Although romantic relationships are not uncommon [46], uncommitted relationships have recently become popular among young people [47]. The inability to commit, associated with breadcrumber perpetrators, may lead to discord between ideal and actual relationship progressions or relationship inactualization, which is associated with poor relationship quality and has strong implications for the mental health of victims [48].

On the other hand, the dependence factor extracted could be explained by relationship-contingent self-esteem [49], since it is believed that breadcrumber perpetrators do so in order to boost their self-esteem (e.g., reasserting themselves, feeling loved and desired by victims) and feeling ownership, knowing that they are not alone but they have their victim “there”.

In short, the internet, as well as the development of computer applications and virtual platforms, has revolutionized the paradigm of couples’ relationships [50]. This cyber society context [51] has changed the way adolescents communicate and manage social, emotional and sexual relationships [52]. It has led to new ways of interacting with others and new virtual means of social interaction [53]. There is evidence that adolescent reliance on social media and the internet for communication and connectivity has both positive and negative affects [24]. Some authors seem to agree on the fact that in some cases, technology exacerbates and facilitates certain forms of victimization in intimate relationships [3,24]. However, there is still debate on about whether digital media simply has introduced a new avenue for more traditional (in-person) unhealthy and abusive behaviors (e.g., harassment, monitoring, and controlling behaviors) or whether these behaviors are new [22,54].

Being aware of the fact that first sexual-affective experiences are critical because they become an important basis of interpretation and behavior in subsequent relationships [55], an understanding of the role played by social media in the formation, maintenance, conflict resolution, and dissolution of teen dating relationships will provide important insights that help fill existing gaps. In agreement with Howard et al. [24], adolescents that experience abusive relationships in their teens carry these unhealthy patterns of abuse into future relationships. Engaging them in the analysis of the benefits and dangers of social media may be instrumental to healthy teen development in the age of technology.

Awareness of the relevance of this phenomenon, adolescents and their families should be provided with education about internet safety behaviors. Health professionals working with adolescents should provide them with knowledge and information about both healthy and unhealthy intimate relationships, and with resources and tools to assist them in navigating social media to develop mentally healthy ways that facilitate their empowerment and prevent harm to themselves or others. Additionally, it is essential to assist young people in adequately identifying digital dating perpetration and provide them with the necessary resources to effectively cope with such victimization. Adolescents need to be encouraged and supported to tell other students or an adult about the drama and discord they witness on social media to prevent it from escalating to more serious complications. The BREAD-ASR questionnaire constitutes a valuable resource that can help health professionals in the screening of breadcrumbing perpetration and in the design of effective prevention and intervention programs in the community.

### Limitations and Further Research

Although the results obtained confirmed the validity and reliability of the designed instrument, the study did show limitations. The instrument developed only perpetration measures; therefore, it would not be appropriate to use it to explore breadcrumbing victimization. The development and psychometric testing of a new instrument to explore breadcrumbing victimization might be considered in future research. The temporal stability of the instrument could not be verified as no test-retest was performed. The psychometric properties obtained for the BREAD-ASR questionnaire could not be contrasted with other studies as it is a new instrument. Similarly, the lack of research on the subject meant that the discussion of the study results was limited. This study was conducted in the Spanish language; therefore, its current application is limited to the Spanish-speaking population. The authors recommend performing confirmatory factors analyses (CFA; to verify construct validity) with criterion validity of different samples in future studies. Thus, further research that expands the samples and settings is necessary. Indeed, the translation and psychometric analysis of this new instrument into other languages is needed in order to apply it in different samples and settings of study. Future research should use the BREAD-ASR to explore the prevalence and impact of the breadcrumbing phenomenon in greater depth and establish a more specific perpetrator profile. This will enable the design of specific prevention and intervention programs that are adapted to successfully managing these toxic behaviors and the potential consequences among the adolescent community.

## 5. Conclusions

The BREAD-ASR questionnaire constitutes a valid and reliable instrument for studying the practice of breadcrumbing. The results of the psychometric properties evaluated show an adequate content validity, construct validity and internal consistency for the questionnaire, making it a valid and reliable instrument that can be applied in future investigations related to the breadcrumbing phenomenon.

## Figures and Tables

**Table 1:** Sociodemographic characteristics of the participants.

Variable	*n*|Mean	%|SD
Sex		
Male	136	55.1
Female	111	44.9
Age	14.53	1.46
Course (Secondary Education)		
First	49	19.9
Second	58	23.5
Third	70	28.4
Fourth	70	28.3
Are you currently in a relationship?		
Yes	98	39.7
No	149	60.3
Have you ever been in a relationship?		
Yes	238	96.4
No	9	3.6
Duration of the relationship (months)	8.09	9.6

**Table 2:** Mean and Standard Deviation of each Item.

Item	Mean	SD
1. When my partner expresses his feelings, I feel uncomfortable and try to end the conversation	1.56	1.07
2. My boyfriend/girlfriend is the reason I have incongruous behavior	1.75	1.12
3. I make my partner feel hopeful that I want a relationship together to excite him/her	1.78	1.21
4. Communication with my partner depends on my interest and availability	2.36	1.42
5. I feel the need to have someone by my side who cares about me	2.65	1.56
6. I feel nervous or uncomfortable when my partner proposes future plans	1.85	1.21
7. I avoid talking with my partner about future plans	2.2	1.37
8. I avoid being in person with my partner	1.36	0.91
9. When I talk with my partner about our future, my answers are unspecific	2.17	1.23
10. I contact my partner when I feel alone	2.61	1.57
11. I communicate with my partner to increase my self-esteem	2.53	1.42
12. I can spend weeks without communicating with my partner	1.57	1.03
13. When my partner contacts me I ignore their messages	1.6	0.97
14. I avoid talking about feelings with my partner	1.87	1.18
15. I always look for an excuse when my partner wants to deepen the relationship	1.94	1.31
16. Most of the interaction with my partner is through social networks	3.89	1.28

**Table 3:** Exploratory factor analysis (EFA) based on the polychoric matrix using principal axis and varimax rotation.

Item	Factor 4	Factor 1	Factor 3	Factor 2	h^2^	u^2^	com
5	**0.64**				0.49	0.50	1.41
1	**0.60**				0.59	0.40	2.15
3	**0.47**				0.33	0.66	2.01
4	**0.36**				0.29	0.71	2.83
2	**0.31**				0.19	0.81	3.07
16	**0.29**				0.13	0.87	2.04
13		**0.79**			0.63	0.37	1.03
15		**0.46**			0.44	0.56	2.86
8		**0.43**			0.38	0.61	2.85
14		**0.41**			0.32	0.68	2.80
6			**0.93**		0.88	0.12	1.03
7			**0.35**		0.21	0.79	2.38
9			**0.27**		0.18	0.82	3.40
10				**0.85**	0.75	0.25	1.09
11				**0.57**	0.44	0.56	1.71
12				**0.28**	0.12	0.88	2.30
Variance	0.11	0.10	0.10	0.09			Mean = 2.2

h^2^ = communality of the item; u^2^ = uniqueness of the item; com = Hoffmann’s item of complexity. Weights lower than 0.25 are hidden; boldface represent correct item-factor weight.

**Table 4:** Factor correlation matrix.

Factors	F1	F2	F3	F4
F1. Communication	1	0.01	0.04	0.15
F2. Avoidance	0.01	1	0.02	0.11
F3. Commitment	0.04	0.02	1	0.06
F4. Dependence	0.15	0.11	0.06	1

Extraction method: principal axis factoring. Rotation method: varimax with Kaiser normalization.

**Table 5:** Fit indexes for the proposed factor model.

Indexes	Result
Root Mean Squared of the Residuals (RMSR)	0.04
Root Mean Squared Error of Approximation (RMSEA)	0.05
Tucker–Lewis Index (TLI)	0.90

**Table 6:** Internal consistency results for the Breadcrumbing Affective-Sexual Relationships (BREAD-ASR) questionnaire.

Item	Average Inter-Item Correlation	Corrected Item-Total Correlation (C-ITC)	Ordinal Alpha If the Item Has Been Deleted
1	0.218	0.567	0.807
2	0.231	0.406	0.818
3	0.225	0.472	0.813
4	0.226	0.479	0.814
5	0.229	0.437	0.817
6	0.230	0.415	0.817
7	0.232	0.385	0.819
8	0.223	0.509	0.811
9	0.232	0.410	0.819
10	0.231	0.388	0.818
11	0.230	0.419	0.817
12	0.239	0.266	0.824
13	0.233	0.353	0.819
14	0.225	0.481	0.813
15	0.219	0.560	0.808
16	0.238	0.346	0.824
17	0.229	0.104	0.826
